# Follistatin Effects in Migration, Vascularization, and Osteogenesis *in vitro* and Bone Repair *in vivo*

**DOI:** 10.3389/fbioe.2019.00038

**Published:** 2019-03-01

**Authors:** Shorouk Fahmy-Garcia, Eric Farrell, Janneke Witte-Bouma, Iris Robbesom-van den Berge, Melva Suarez, Didem Mumcuoglu, Heike Walles, Sebastiaan G. J. M. Kluijtmans, Bram C. J. van der Eerden, Gerjo J. V. M. van Osch, Johannes P. T. M. van Leeuwen, Marjolein van Driel

**Affiliations:** ^1^Department of Orthopedics, Erasmus MC, University Medical Center, Rotterdam, Netherlands; ^2^Department of Internal Medicine, Erasmus MC, University Medical Center, Rotterdam, Netherlands; ^3^Department of Oral and Maxillofacial Surgery, Erasmus MC, University Medical Center, Rotterdam, Netherlands; ^4^Institute of Tissue Engineering and Regenerative Medicine, Julius-Maximillians University Würzburg, Würzburg, Germany; ^5^Fujifilm Manufacturing Europe B.V., Tilburg, Netherlands; ^6^Department of Otorhinolaryngology, Head and Neck Surgery, Erasmus MC, University Medical Center, Rotterdam, Netherlands

**Keywords:** follistatin 315 (FST315), follistatin 288 (FST288), migration, vascularization, osteogenesis, injectable *in situ* gelling slow release system, bone tissue engineering, regenerative medicine

## Abstract

The use of biomaterials and signaling molecules to induce bone formation is a promising approach in the field of bone tissue engineering. Follistatin (FST) is a glycoprotein able to bind irreversibly to activin A, a protein that has been reported to inhibit bone formation. We investigated the effect of FST in critical processes for bone repair, such as cell recruitment, osteogenesis and vascularization, and ultimately its use for bone tissue engineering. *In vitro*, FST promoted mesenchymal stem cell (MSC) and endothelial cell (EC) migration as well as essential steps in the formation and expansion of the vasculature such as EC tube-formation and sprouting. FST did not enhance osteogenic differentiation of MSCs, but increased committed osteoblast mineralization. *In vivo*, FST was loaded in an *in situ* gelling formulation made by alginate and recombinant collagen-based peptide microspheres and implanted in a rat calvarial defect model. Two FST variants (FST288 and FST315) with major differences in their affinity to cell-surface proteoglycans, which may influence their effect upon *in vivo* bone repair, were tested. *In vitro*, most of the loaded FST315 was released over 4 weeks, contrary to FST288, which was mostly retained in the biomaterial. However, none of the FST variants improved *in vivo* bone healing compared to control. These results demonstrate that FST enhances crucial processes needed for bone repair. Further studies need to investigate the optimal FST carrier for bone regeneration.

## Introduction

Biomaterial scaffolds functionalized to stimulate endogenous repair mechanisms incorporating bone-forming factors offer a potential alternative to bone-grafts. Furthermore, injectable systems are a promising option since they can potentially deliver growth factors in a less invasive manner and conform to complex shapes, which is especially important within the craniofacial complex (Kretlow et al., 2009). Therefore, choosing the right material and growth factor(s) is critical to induce the key events needed for bone formation. In previous work, we have developed an *in situ* gelling formulation made by alginate and Recombinant Collagen-based Peptide (RCP) microspheres (MS) that were able to considerably slow down the release of bone morphogenetic protein 2 (BMP-2) and support bone formation (Mumcuoglu et al., 2018). However, despite the therapeutic potential of BMP-2 in bone repair, it is not free of complications such as ectopic bone formation, respiratory failure, inflammation, pseudarthrosis, and cancer (Poon et al., 2016).

The TGF-β family comprises more than 30 signaling proteins that are essential developmental factors stimulating diverse cellular differentiation and growth responses (Hinck and Huang, 2013). Among them, bone morphogenetic proteins (BMPs) and the activin/inhibin subfamily members such as BMP-2 or activins have been shown to be fundamental in the regulation of bone organogenesis (Abe et al., 2004b; Rahman et al., 2015). Particularly, activin A is one of the most abundant TGF-β family member proteins found in bone (Pearsall et al., 2008). Consequently, bone contains high concentrations of BMPs and activins as well as their antagonists such as noggin or follistatin (FST), which block signaling and are essential regulators of endogenous bone repair. Their importance is such that Matzuk et al. demonstrated that follistatin-deficient mice were growth-retarded and among others showed skeletal defects, dying within hours of birth (Matzuk et al., 1995). The effect of FST on bone repair and the major target it antagonizes, activin A, is not clear. It has been previously reported that activin A stimulates osteoclast formation (Sakai et al., 1993; Koseki et al., 2002), and that the administration of soluble activin receptors enhances bone formation and bone mineral density in both mice and monkeys (Pearsall et al., 2008; Fajardo et al., 2010; Bialek et al., 2014).

In bone, FST is mainly expressed by osteoblasts, but also to a lesser extent by osteocytes in both developing mouse mandible and in the callus of repairing bone (Inoue et al., 1994). Its role in osteogenic differentiation is controversial, both a positive and a negative effect upon osteoblasts during osteogenic differentiation have been reported (Abe et al., 2004a; Eijken et al., 2007). *In vivo*, several studies have used follistatin-overexpressing mice to assess its effect upon muscle healing and bone morphology, concluding that FST improved muscle healing after injury, but its overexpression leads to a decreased quality of the skeleton (Zhu et al., 2011; Gajos-Michniewicz et al., 2012). Kawao et al. have demonstrated that in response to hyper gravity, FST acts as a circulating molecule regulating muscle and bone metabolism and the interaction between them (Kawao et al., 2017). Taken together, these data suggest that FST administration, as the major antagonist of activin A, is involved in bone formation and may have a significant therapeutic potential to trigger bone regeneration.

The role of follistatin in some of the other essential processes needed for bone repair such as cell recruitment or vascularization is suggested but remains unclear. FST has been associated with angiogenin, a key protein implicated in activation of endothelial cells and stimulation of new blood vessel growth (Gao et al., 2007). Nonetheless, whether FST promotes angiogenesis is uncertain. Some studies have shown that FST induces tumor-associated angiogenesis (Kozian et al., 1997; Krneta et al., 2006), but FST was also found to inhibit it (Ogino et al., 2008).

Follistatin is a structurally complex monomeric glycoprotein widely distributed throughout adult tissues (Patel, 1998). The FST gene consists of six exons with an alternative splicing site that generates two major forms and, after further proteolysis and glycosylation, results in two different variants of the protein. FST288, which consists of 288 amino acids and FST315, which consists of 315 amino acids (Phillips, 1998). The FST315 variant includes an acidic C-terminal tail domain encoded by exon 6, whereas the FST288 protein ends after exon 5 due to a stop codon inserted as a result of alternative splicing. Importantly, although both FST variants contain a heparin-binding sequence, which affords an ability to bind to cell surface proteoglycans on many cells, the C-terminal domain in FST315 seems to neutralize the basic residues of the heparin binding sequence (Schneyer et al., 2004). Thus, FST315 can only bind to proteoglycans after binding to activin, which causes a conformational change that exposes the heparin-binding motif (Hedger et al., 2011). Therefore, in general FST315 exhibits weak cell-surface binding capability and is considered the circulating form of the protein, while FST288 is the tissue-bound variant due to its ability to bind to heparan-sulfate proteoglycans (Patel, 1998; Gajos-Michniewicz et al., 2010). The structural differences between both follistatin variants may have an impact on their properties and ultimately their regulatory role. In fact, several groups have studied the pharmacokinetic/pharmacodynamic (PK/PD) relationships and their effect in muscle regeneration (Datta-Mannan et al., 2013; Yaden et al., 2014) but unfortunately, most of the published studies did not specify which FST variant was used (Abe et al., 2004a; Eijken et al., 2007; Zhu et al., 2011; Bowser et al., 2013; Kawao et al., 2017).

No receptor for FST has been found but it is known to bind almost irreversibly to activin A, neutralizing its function (Sidis et al., 2001; Schneyer et al., 2003). Both variants of FST inhibit activin A function, although it has been shown that the affinity of FST288 for activin A is higher than that of FST315 (Hashimoto et al., 2000). Also, FST is able to interact with other members of the TGF-β superfamily neutralizing or regulating their functions such as activin B, myostatin and BMPs. With 10-fold lower affinity than for activin A, FST can also bind to BMP4, 6, and 7 (Glister et al., 2004; Sidis et al., 2006; Cash et al., 2012). As bone metabolism is regulated mostly by BMPs and activin A, follistatin seems to play a pivotal role in bone physiology.

This study has been performed to investigate the use of FST for bone tissue engineering. *In vitro*, we have investigated whether FST is able to attract osteoprogenitor and endothelial cells from human origin and promote their differentiation. We then assessed the effect of FST in bone regeneration in a rat calvarial defect model and loaded two different doses of both variants of FST—FST315 and FST288—in our previously developed controlled-delivery system of RCP encapsulated in an injectable alginate hydrogel (Mumcuoglu et al., 2018).

## Materials and Methods

### Cell Culture

Human MSCs were obtained from leftover material from iliac crest biopsies of four juvenile donors (age 9–12 years) undergoing cleft palate reconstruction surgery with implicit consent (Erasmus MC medical ethical committee number MEC-2014-106) and expanded as previously described (Fahmy-Garcia, 2017). Then, cells at passage 4 were used either for migration or osteogenic differentiation assays.

Primary microvascular endothelial cells (MVECs) were isolated from the foreskin of three juvenile donors (age 9 mos-4 years) as previously described (Kleinhans et al., 2013) after their guardians had provided full informed consent (University of Wurzburg ethical board vote 182/10). Then, cells were cultured as monolayers in endothelial cell growth medium MV (ECGM; PromoCell). Cells were passaged at 70–80% confluence and were used between passages 3 and 5 for sprouting assays.

HUVECs (Lonza, Walkersville, MD, USA) were cultured at a density of 5,000 cells/cm^2^ in endothelial growth medium [EGM-2 with SingleQuots (Lonza)]. Non-adherent cells were removed by replacing the medium after 2–3 days. When cells neared confluency they were used between passages 9 and 12 either for migration or tube formation assays.

Calvarial-derived Simian Virus-immortalized Human Fetal Osteoblast (SV-HFO) cells (Chiba et al., 1993) were expanded and cultured as published previously (Eijken et al., 2006). Briefly, cells were seeded in phenol-red free α-Minimal Essential Medium (a-MEM Gibco, BRL, Paisley, UK), pH 7.5, supplemented with 20 mM HEPES (Sigma, St. Louis, MI), penicillin/streptomycin, 1.8 mM CaCl_2__2H_2_O (Sigma) and 2% heat-inactivated charcoal-treated fetal calf serum (FCS). All cell cultures were performed in 5% CO_2_ at 37°C in a humidified atmosphere.

### Migration Assay

The effect of FST upon MSC and HUVEC migration was assessed using modified Boyden chambers (polyethylene terephthalate cell culture inserts, pore size: 8 μm in diameter, Millipore-Merck, NL). The human recombinant FST variants used in this study were made via CHO-S cell expression using the FST's native leader sequences, followed by affinity protein chromatography using Hi-trap heparin (GE Healthcare Life Science), and desalted to a 50 mM KPO_4_ buffer (165 mM sucrose, 0,01% Tween-20, pH 7.4). SDS-PAGE showed a protein purity higher than 95%.

To analyze MSC migration, α-MEM (Gibco) containing FST315 [0.8–5 nM (28–175 ng/ml)] was added to the lower chamber of a 24-well-plate. The doses selected were based in a previous publication, in which 100 and 500 ng/ml of FST were used, showing that these range of doses was able to enhance osteoblast osteogenic differentiation (Eijken et al., 2007), and in previous non-published studies carried out in our department in which lower FST doses were tested. αMEM containing PDGF-AB (20 ng/ml) was used as positive control. The controls used to investigate MSC migration were also used as controls in another publication (Fahmy-Garcia, 2017). 6 × 10^3^ MSCs suspended in a volume of 200 μl α-MEM were added to the upper chamber. Cell migration was followed at 5% CO_2_ and 37°C in a humidified atmosphere for 17 h.

To test HUVEC migration, endothelial cell basal medium (EBM-2, Lonza) containing FST was added to the lower chamber. 5 × 10^4^ HUVECs were added into the upper chamber of the transwells and incubated at 5% CO_2_ and 37°C in a humidified atmosphere for 10 h. EGM-2 medium was used as positive control. The membrane was then washed and the cells were fixed with 4% formalin and stained with 4′, 6-diamidino-2-phenylindole (DAPI) (100 ng/ml) in the dark for 5 min. Cells remaining on the upper surface of the membrane were mechanically removed with a cotton swab and those which that had migrated to the lower surface were imaged using fluorescence microscopy (Zeiss Axiovert 200M Fluorescence Imaging, Sliedrecht, NL) in five random fields for each membrane and counted using ImageJ software.

### HUVEC Tube Formation Assay

Growth factor-reduced matrigel (Corning, USA) was added to a 96-wells plate and incubated at 37°C for 1 h. HUVECs were trypsinized and resuspended in EBM medium supplemented with FST315 2-fold concentrated to achieve 28, 70, and 175 ng/ml as final concentrations. EGM-2 complete medium was used as positive control and EBM-2 medium as negative control. 15,000 Cells were seeded on top of the matrigel and incubated at 37°C in the presence of 5% CO_2_ in a humidified atmosphere. Tube formation was imaged after 4, 6, and 24 h of incubation. The results were analyzed using ImageJ software.

### 3D-Culture Spheroid Assay

To generate the spheroids, 250,000 MVECS were suspended in 40 ml of Vasculife® complete medium (Lifeline, Germany) with 10 ml of methyl cellulose [MethocelTM (Dow, USA)], seeded in non-adherent round-bottom 96-well-plates (100 μl/well) and incubated overnight at 37°C and 5% CO_2_ in a humidified atmosphere. The following day spheroids (500 cells approx. per spheroid) were harvested, transferred to a 50 ml tube and centrifuged (3 min, 500 g). Supernatant was removed and the pellet was covered with 5 ml of Methocel™. Immediately prior to use, collagen preparation was made by 3.3 ml pepsin-extracted type I collagen (Rat tail collagen High Concentration, type 1, BD Biosciences, USA) solubilized in 1.7 ml 0.1% acetic acid and 0.5 ml Medium 199 (10x) (Sigma). 0.5 Ml of NaOH 0.2 N was used to reach a neutral PH. Collagen preparation was mixed with the spheroid-containing methocel solution and seeded in 24-well-plates (1 ml/well). Plates were placed into an incubator to let the collagen gel polymerize for 30 min. Then, collagen gels were overlaid with 200 μl medium containing FST315 10-fold concentrated to achieve a final concentration of 28 ng/ml. VEGF at 27.5 ng/ml was used as positive control and basal medium as negative control. After 24 h of culture, cell invasion was visualized using a ZEISS Axiovert 25 microscope at 10x magnification.

### Osteogenic Differentiation

Osteogenic differentiation assays were performed on MSCs and SV-HFOs. 3000 cells/cm^2^ (MSCs) or 9000 cells/cm^2^ (SV-HFOs) were seeded in α-MEM in 12-well-plates. For MSCs, the medium was replaced after 24 h with complete osteogenic medium; DMEM High Glucose (Gibco) with 10% FCS, 1.5 μg/mL fungizone, 50 μg/mL gentamicin, 25 μg/mL ascorbic acid-2-phosphate, 10 mM β-glycerophosphate, and 0.1 μM dexamethasone. For SV-HFOs, medium was replaced after 48 h with osteogenic differentiation medium consisting of phenol-red free α-MEM (Gibco) supplemented with 20 mM HEPES (Sigma, MO, USA), streptomycin/penicillin, 1.8 mM CaCl_2_·2H_2_O (Sigma), 2% heat-inactivated charcoal-treated FCS, 0.1 μM dexamethasone and 10 mM β-glycerophosphate. To both cultures FST315 (28, 70, and 175 ng/ml) was added during each medium refreshment. The experiment was carried out until onset of mineralization, monitored by measuring calcium concentration in the culture supernatant. For biochemical analyses, medium was collected and cells were scraped from the culture dish in PBS containing 0.1% Triton X-100. Supernatants were stored at −80°C. Cell lysates were thawed and sonicated on ice in a sonifier cell disruptor (Soniprep 150, MSE, London, UK) or in a water-bath sonifier (Ultrasonic Cleaner CD-4800, Norville, UK) before analysis.

#### Alkaline Phosphatase (ALP) Activity and Protein Measurement

ALP activity was performed as described previously (Lowry et al., 1954). Briefly, it was assayed by determining the release of paranitrophenol from paranitrophenylphosphate (pNPP) in the SV-HFO cell lysates as previously described (Fahmy-Garcia, 2017). Absorption was measured on the Wallac 1420 Victor2 plate reader at 405 nm. For standards, ALP (10 U/ml) from bovine kidney (Sigma) was used.

Protein was measured in cell lysates using the BCA protein assay (Pierce™ BCA Protein assay, Thermo Scientific, Rockford, IL) according to manufacturer's instructions.

#### Mineralization

To quantify the calcium content, cell lysates were incubated for 48 h in 0.24 M HCl at 4°C. For analysis of calcium concentration in the culture medium, supernatant was collected and measured directly from day 9 onwards. In both cases, calcium content was colorimetrically determined after addition of 1M ethanolamine buffer (pH 10.6), 19.8 mM 8-hydroxyquinoline and 0.35 mM 0-cresolphtalein complexone, at 595 nm on the Wallac 1420 Victor2. Besides, to assess that calcium deposition was, in fact, observed due to the osteogenic differentiation of the cultured cells and not just due to a mere calcium precipitation, MSCs and SVHFOs were also cultured in osteogenic medium but in the absence of dexamethasone. The results showed that, without the addition of dexamethasone, calcium deposition was not detectable (Supplementary Figure 1).

For von Kossa staining, cell cultures were fixed for 15 min in 4% formaldehyde. After fixation, cells were washed five times with distilled water. Subsequently, calcium was stained by 5% (w/v) silver nitrate solution (Sigma, 85228) for 30 min under a 60 W light. Next, the culture plate was rinsed with distilled water and dehydrated with ascending concentrations of ethanol (70, 96, and 100%). Subsequently, ethanol was removed, and cell culture plates were air-dried and imaged using inverted microscope (Olympus CKX41, Zoeterwoude, NL).

#### Quantification of Follistatin and Activin A

Quantification of both human FST and human activin was measured using the follistatin DuoSet ELISA kit and the activin A Duoset ELISA kit (R&D Systems). Briefly, 48–72 h after replacement, conditioned medium was collected until onset of mineralization and stored at −80°C. The ELISAs were performed according to the manufacturer's protocol.

### Preparation of the Hydrogel Formulation for Protein Release and *in vivo* Studies

Recombinant Collagen Peptide (RCP) Microspheres (MS) with an average size of 50 μm were produced by emulsification using calcium carbonate (CaCO_3_) as described previously (Mumcuoglu et al., 2017). Pronova SLG20 (sterile alginate where over 60% of the monomer units are guluronate) was ordered from Novamatrix (Sandvika, Norway) and was dissolved in 0.9% sterile sodium chloride to create 2% w/v solution.

Thirty-four milligram of calcium containing microspheres were incubated overnight at 4°C with FST315, FST288, or BMP-2. The recombinant human bone morphogenetic protein-2 (rhBMP-2, amino acids 283–396 plus an N-terminal Met-Ala) was expressed in *Escherichia coli*, isolated from inclusion bodies, renatured and purified, as previously described (Kirsch et al., 2000), and it was kindly provided by Dr. Joachim Nickel (Fraunhofer IGB, Germany). For release assays, 85 μl of FST288 or FST315 at a concentration of 112.5 μg/ml was added to the MS to achieve a final concentration of 1.48 μg in the final formulation. For *in vivo* studies, FST288 and FST315 at 152 μg/ml and 15.2 μg/ml were added overnight to the MS to achieve a concentration of 20 and 2 μg/ml. Eighty-five microliter of 380 μg/ml BMP-2 was added to the MS, resulting in a concentration of 50 μg/ml within the final formulation. After overnight incubation, on top of swollen particles, 507 μl of SLG was added. Then, the mixture was supplemented with 53 μl of 0.06 M fresh glucono delta lactone (GDL) solution (Sigma). GDL was used to dissolve the CaCO_3_ so that alginate could be crosslinked and increase the mechanical property of the formulation. The prepared formulation was incubated overnight at 4°C to equilibrate. Next day, the formulation was mixed again prior to *in vitro* and *in vivo* studies.

#### Release of FST315 and FST288 From the Hydrogel Formulation

The formulations were prepared as described above; 100 μl from the hydrogel with 1.48 μg of either one of the both variants of FST was added to each well of 24-well-plate inserts with 0.4 μm pore size. One milliliter DMEM with 10% FBS and 1% Penicillin/Streptomycin per well was added to reservoir plate. The plates were incubated at 37°C under constant agitation at 300 rpm. At each time point the medium was collected and replaced with fresh medium. The collected release media was analyzed by duoset FST ELISA kit (R&D) according to manufacturer's protocol. As positive control, 100 μl of 1.48 μg/ml FST288 and FST315 solution were added to the inserts without hydrogel constructs and 1 ml medium was added to bottom wells of the transwell plate. At each time point 1 ml medium was collected and changed with fresh medium.

### *In vivo* Study

All animal experiments were performed with prior approval of the ethics committee for laboratory animal use (protocol #EMC 116-15-04).

Twenty-six Sprague Dawley (SD) male rats (Envigo, NL) at 12 weeks old were used in this study to evaluate bone formation. The animals were randomly assigned and housed in pairs in a specific pathogen-free environment and allowed to adapt to the conditions of the animal house for 7 days before starting the study. The animals were maintained at 20–26°C on a 12 h dark/light cycle with *ad libitum* access to standard rat chow and water. To evaluate the effect of FST288, FST315, and BMP-2, the proteins were loaded in the alginate formulation. Forty microliter of the protein loaded-composite was injected in the defect. Two different concentrations of FST315 and FST288 were loaded in the defects, 800 and 80 ng (*n* = 9 defects). BMP-2 was used as positive control and the concentration loaded per defect was 2 μg (*n* = 3 defects). As negative control, biomaterials without FST were implanted (*n* = 10 defects). Each animal received two implants in bilateral defects. Animals were euthanized with CO_2_ and the specimens were harvested for further analysis 10 weeks after implantation. The biomaterial only control used in this experiment was also used as control in another publication (Mumcuoglu et al., 2018) to reduce the number of experimental animals.

#### Surgical Procedure and Fluorochrome Labeling

The rat calvarial defect was performed as previously described (Spicer et al., 2012). After general anesthesia using 2.5% isoflurane, the animals received intraperitoneal injections of 0.05 mg/kg of buprenorphine (Temgesic®, Indivior, UK) for perioperative analgesia and 5 ml/kg sterile normal saline to account for fluid losses. The animal skulls were shaved and disinfected with ethanol swabs. Then, an incision was made through the skin of the calvarium and periosteum, and full-thickness flaps were reflected. The defect was irrigated with 0.1 ml of 1% xylocaine with 1:200,000 epinephrine (AstraZeneca, NL) along the sagittal midline of the skull. Under copious sterile saline irrigation, two 5-mm-diameter bone defects were prepared with a trephine bur (Fine Science Tools, Germany) in each animal and any debris or bone chips were removed. The defects were treated with the biomaterial loaded with FST, BMP-2 or the biomaterial alone as described above. Then the periosteum and the skin over it were repositioned and sutured with polylactic acid sutures (Vycril 4.0, Ethicon, Johnson Prod., São José dos Campos, Brazil). All animals received three postoperative doses of buprenorphine for analgesia every 10 h during the next days. Four weeks postoperative, rats were subcutaneously injected with 25 mg/kg of Calcein (Sigma) in a 2% sodium bicarbonate solution. Fluorochrome label was analyzed using a light/fluorescence microscope with a filter block (Zeiss Axiovert 200M Fluorescence Imaging, Sliedrecht, NL).

#### Micro-CT Analysis

Quantum FX micro-CT (Perkin Elmer, Waltham, MA, USA) was used to image animals biweekly until the end of the experiment. To image the bone formation *in vivo* the following parameters were used; Field of view: 30 mm, Voltage: 90 kV, Current: 160 μA, Scan Time: 3 min. To image the implants after retrieval, a field of view of 20 mm and a scan time of 4.5 min were used. Mineral volume and bone mineral density (BMD) were measured on basis of calibration scanning, using two phantoms with known density (0.25 and 0.75 g/cm^3^; Bruker Micro-CT) under identical conditions. For image processing, analysis software was used (Mayoclinic, Rochester, MN, USA), threshold levels were set to 0.12 g/cm^3^.

#### Histological Evaluation

Ten weeks after implantation, the relevant part of the skull was removed and fixed in neutral buffered 4% formalin solution for 3 days, dehydrated in graded ethanol solution from 70 to 100%, and finally embedded in methyl methacrylate resin. Sections of 10 μm were generated along the long axis of the cylindrical samples on a saw Microtome system (Leica 4 SP1600, Germany). Samples were stained with von Kossa and Goldner's trichrome as previously described (Gruber and Gruber, 1992; Fahmy-Garcia, 2017).

### Statistical Analysis

Data were analyzed with IBM Statistics 21 (SPSS) and GraphPad software (GraphPad, San Diego, USA). Migration, osteogenesis and angiogenesis were analyzed using a linear mixed model; the different conditions (different doses of the proteins studied) were considered a fixed parameter and the donors (experiments) as a random factor. *Ex vivo* data were also analyzed using a linear mixed model. Data are presented indicating the mean ± SD and a value of *p* < 0.05 was considered to be statistically significant.

FST release data were analyzed using a Student's *T*-test, while *in vivo* micro-CT data were analyzed using two-way analysis of variance. If the overall differences were significant, differences between groups were analyzed by Bonferroni *post hoc* test. Data are presented indicating the mean ± SEM.

## Results

### Follistatin Attracts Both MSC and HUVEC Cells *in vitro*

**Figure 1:**
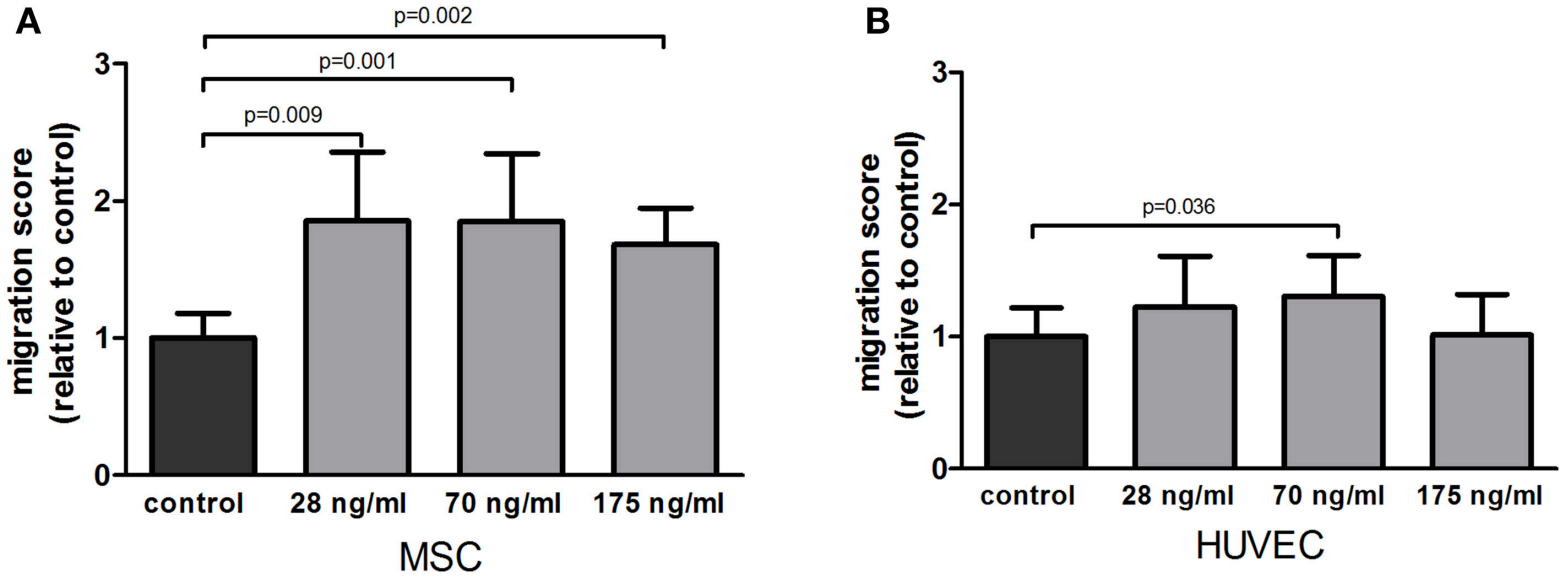
Effect of follistatin upon MSC and HUVEC in a migration assay. **(A)** Average migration of MSCs exposed to several doses of follistatin (FST315) relative to the negative control (*n* = 4 donors in duplicate). **(B)** Average migration of HUVECs exposed to several doses of follistatin (FST315) relative to the negative control (*n* = 3 independent experiments in duplicate). The bars represent the mean ± SD.

To determine if follistatin could recruit osteoprogenitor cells and endothelial cells to the site of injury, we assessed its ability to attract MSCs and HUVECs. FST significantly stimulated MSC migration compared to plain medium control at all doses tested; 1.68, 1.857, and 1.581-fold increase, respectively (*p* = 0.009, *p* = 0.001, *p* = 0.002) (Figure 1A). Migration of HUVECs toward FST-containing medium was less pronounced than in MSCs, and only reached significance at the medium dose (1.28-fold increase of migration compared to control, *p* = 0.036) (Figure 1B).

### Follistatin Promotes Neovascularization and Angiogenesis *in vitro*

**Figure 2:**
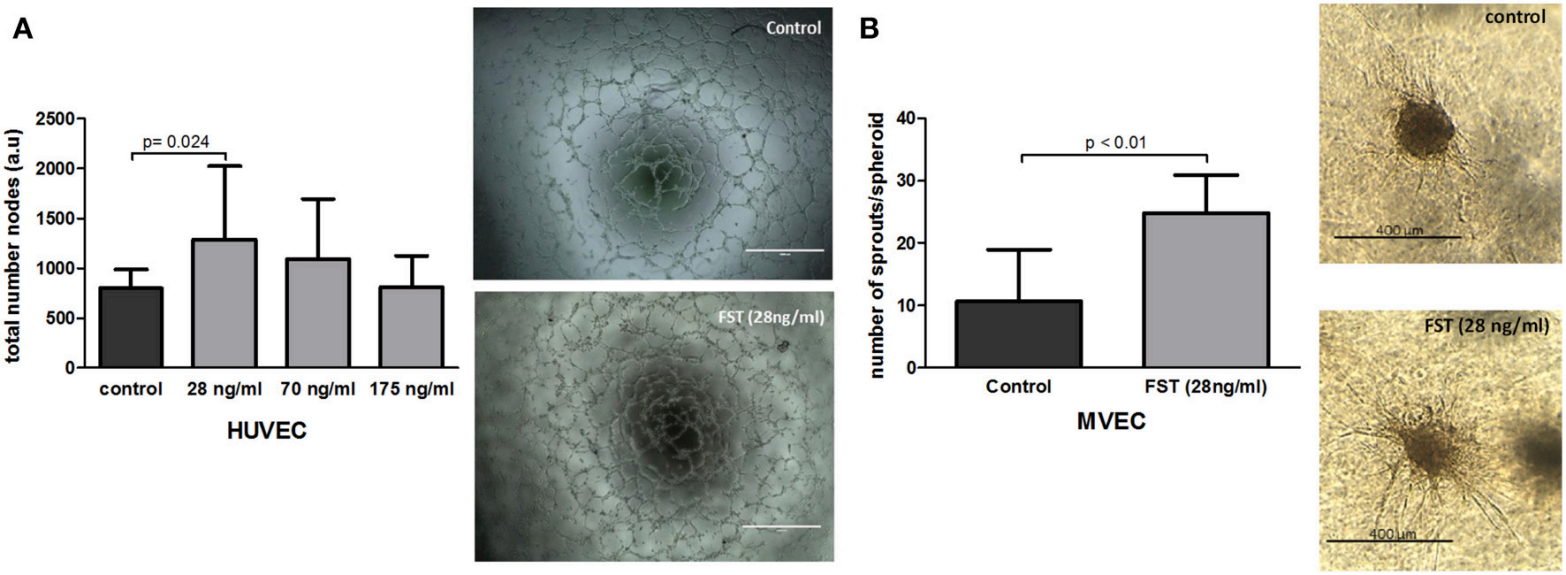
Effect of follistatin on neovascularization. **(A)** Effect of follistatin (FST315) on vasculogenesis. Total number of nodes was quantified (*n* = 3 experiments in triplicate). Next to the graph, representative pictures of tube-like structures are shown after 6 h incubation in the presence and absence of FST (scale bar: 1,000 μm). **(B)** Effect of FST315 on angiogenesis. MVEC spheroids were embedded in collagen and incubated for 24 h. The total number of sprouts per spheroid w/o the addition of FST at 28 ng/ml dose are plotted in the graph (*n* = 10 individual spheroids per experimental group). Next to the graph, representative pictures of cell spheroids in the presence and absence of FST are shown after 24 h incubation (scale bar: 400 μm). The bars show the mean ± SD.

Formation and expansion of the vasculature within the newly formed tissue are the crucial steps in neovascularization, and therefore, in bone repair. Several *in vitro* assays were performed to mimic the different steps of the vascularization process. To determine the capability of FST to promote vasculogenesis *in vitro*, HUVECs were seeded on matrigel coated plates in the absence or presence of FST. FST significantly triggered the formation of tube-like structures in an inverse dose-dependent manner, showing 1.6-fold increase when the lowest dose was supplied compared to the basal medium (*p* = 0.014) (Figure 2A). As positive control, endothelial cells were also treated with EGM-2 complete medium. EGM-2 medium stimulated formation of tube-like structures 1.7 times more than the basal medium (data not shown).

To assess whether 28 ng/ml of FST is also able to induce sprouting angiogenesis, spheroid-sprouting assays were done using MVECs. The spheroid serves as starting point for the growth of capillary-like sprouts. FST stimulated the formation of endothelial cell sprouts from the spheroids, showing more than 2-fold increase in the number of sprouts compared to control (Figure 2B) and with a similar potency to VEGF (data not shown). In summary, these data indicate that FST is able to stimulate the distinct mechanisms involved in the formation of the vascular network.

### Follistatin Does not Stimulate MSC Osteogenic Mineralization, but Stimulates Committed Osteoblast Mineralization

**Figure 3:**
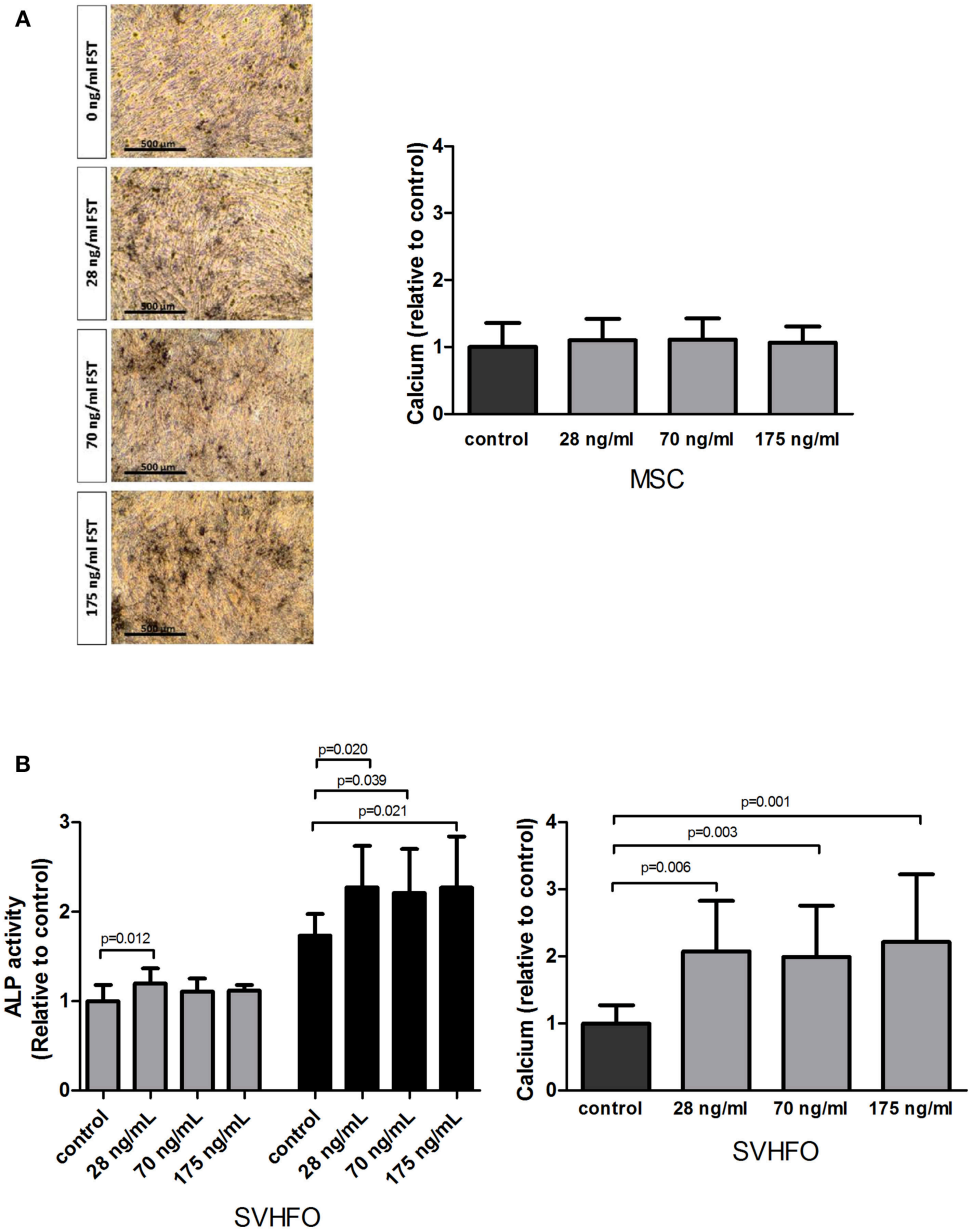
Effect of follistatin on osteogenic differentiation. Human MSCs and osteoblasts were induced to mineralize in the absence or continuous presence of follistatin (FST315). **(A)** Quantification of calcium deposition (nmol/ cm^2^) in the MSC extracellular matrix at the onset of mineralization relative to control (osteogenic differentiation medium) (*n* = 4 donors performed in triplicate). Donor dependently, mineralization started between 18 and 22 days of culture. Representative pictures of the Von Kossa staining at the onset of mineralization (scale bar: 500 μm). **(B)** Left graph: alkaline phosphatase (ALP) activity (mU/ cm^2^) during SV-HFO culture with and without continuous FST treatment at day 9 (gray bars) and 16 (black bars) of culture. Results are shown relative to day 9 control. Right graph: Quantification of calcium deposition (nmol/ cm^2^) in the SV-HFO extracellular matrix at day 16 relative to control (*n* = 3 experiments performed in triplicate). The bars show the mean ± SD.

The osteogenic properties of FST were studied in MSCs stimulated toward the osteoblastic lineage as well as in fetal calvarial-derived committed pre-osteoblasts (SV-HFO). The addition of FST did not affect mineralization of MSCs, as shown by quantitative measurement of calcium deposition and von Kossa mineral staining (Figure 3A). Interestingly, adding FST to the pre-osteoblast culture had a direct positive effect on the mineralization process at any of the tested doses, showing a minimum of 2-fold increase compared to the control (osteogenic differentiation medium) (Figure 3B).

Additional experiments also indicated a positive effect of FST on committed osteoblast differentiation, as is shown via activity of the enzyme alkaline phosphatase. Alkaline phosphatase (ALP) is expressed during osteoblastic differentiation and plays a key role in bone mineralization (Wennberg et al., 2000). When the lowest dose of FST was applied to the pre-osteoblast culture, a small but significant increase in ALP activity was observed before mineralization started (day 9). At day 16, FST enhanced ALP activity in all concentrations tested (Figure 3B).

### Follistatin Production Changes During Osteogenic Differentiation

**Figure 4:**
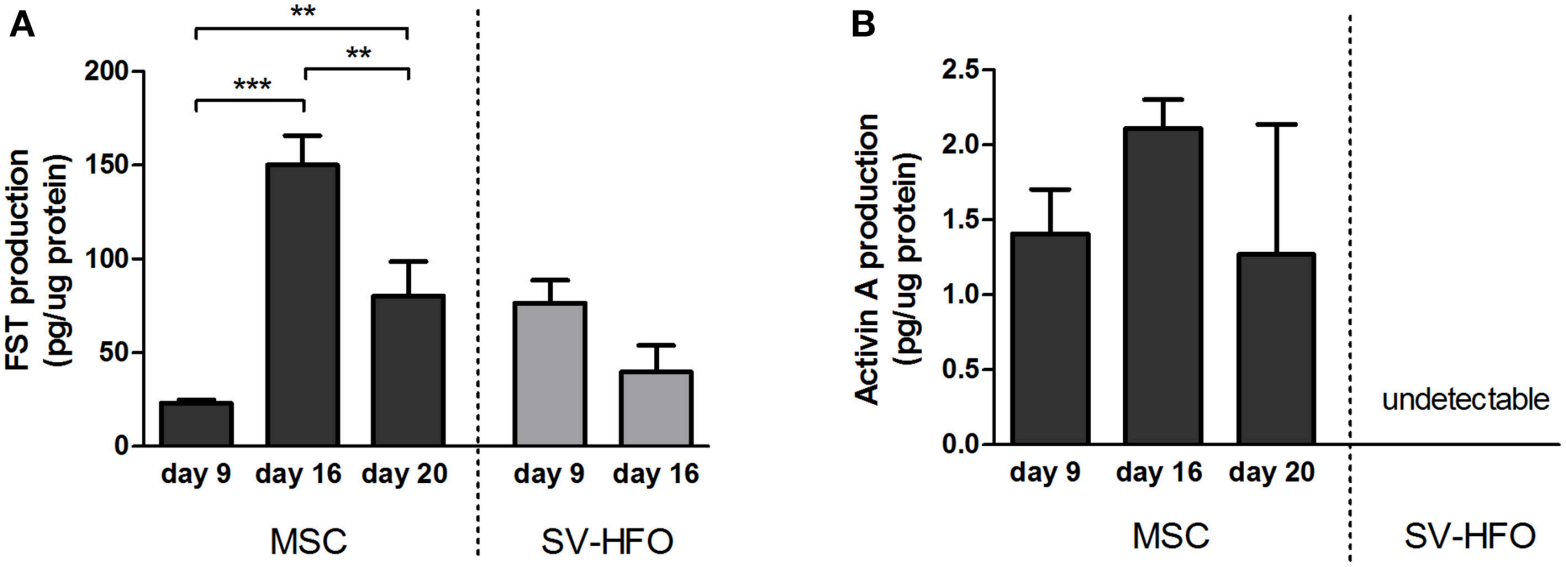
Production of follistatin and activin A by MSCs and osteoblasts. FST **(A)** and activin A **(B)** levels were measured in supernatant of MSC and SV-HFO that were induced to mineralize until onset of mineralization. Production was corrected for cell lysate protein content. The bars show the mean ± SD. **p < 0.01 and ***p < 0.001.

To regulate cellular processes, such as extracellular matrix mineralization, two follistatin molecules encircle activin, neutralizing its receptor binding sites (Thompson et al., 2005). We measured the levels of FST and activin produced during the different phases of osteogenic differentiation in MSC and SV-HFO cultures by ELISA. FST was produced in high quantities by MSCs at the onset of mineralization (day 16) and its release significantly decreased during full mineralization (Figure 4A). Activin production levels did not significantly differ during MSC culture, but remained low (at least 15 times lower than the FST levels at the same time points) (Figure 4B). Unlike MSCs, SV-HFOs are already osteogenic–committed cells and therefore, mineralization occurs earlier than in MSCs. In SV-HFOs, the production of FST decreased from day 9 onwards during osteogenic differentiation (Figure 4A), a similar pattern to what was observed in MSCs, though earlier in culture. Activin levels were undetectable in SV-HFO cultures.

### FST288 Release Is Lower Than FST315 Release From the Alginate-MS Hydrogel

**Figure 5:**
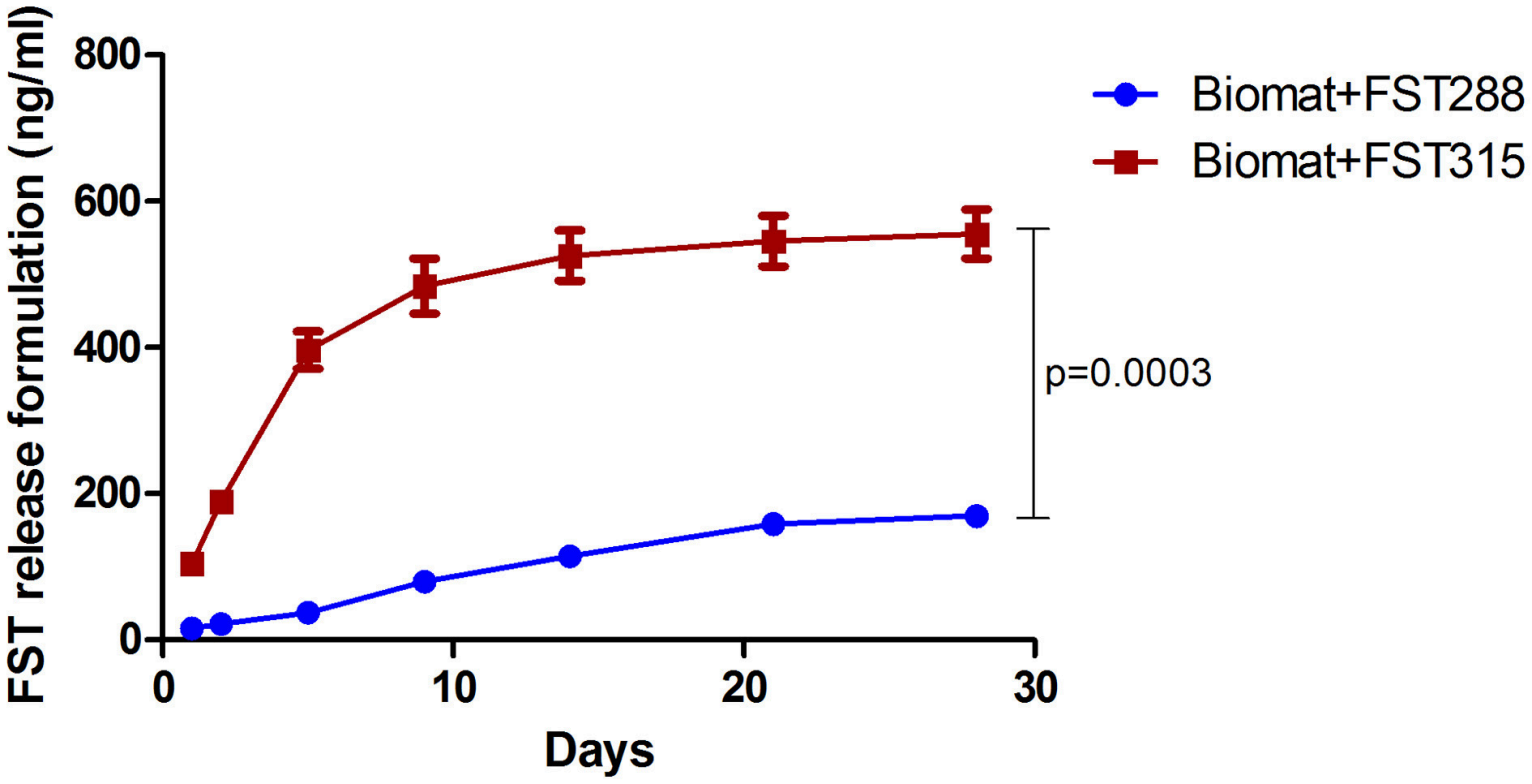
*In vitro* release of FST288 compared to FST315. The cumulative release of FST288 and FST315 from the alginate-MS formulation in DMEM with 1% P/S detected by ELISA is demonstrated over 4 weeks. Data are presented indicating the mean ± SEM. Statistical difference at 28 days of cumulative release analyzed by Student's *t*-test, *p* < 0.001.

It is generally believed that controlled-release systems are optimal for bone formation as they offer spatiotemporal control to mimic the native healing cascades. The previously developed alginate-MS hydrogel was shown to provide sustained protein release (Mumcuoglu et al., 2018). We first evaluated the release profile of FST288 and FST315 from this formulation. After 4 weeks, the cumulative amount of FST315 and FST288 found in the medium was quantified. To correct for the effect of protein sticking to the plate and membrane, as well as its degradation over time, 1.48 μg/ml of FST288 and FST315 were added to the upper part of the transwells without the hydrogel formulation. For FST288 671.60 ± 220.6 ng (mean ± SEM) and for FST315 688.5 ± 78.2 ng (mean ± SEM) were detected, meaning that both FST variants have a similar degradation rate. There are important differences between both FST variants; unlike FST288, FST315 has a C-terminal tail containing several acidic residues, which decrease its heparin affinity. Most likely this difference might affect the release profile of the FST variants. In fact, when the release of both FST variants from the alginate-MS formulation was studied over 4 weeks, significant differences were observed between the release of FST315 and FST288. 555 ± 58 Ng of FST315 was released from the formulation, while only 169.4 ± 6.8 ng of FST288 was released during the same period. Therefore, the amount of FST315 exuded to the medium was three times more than the amount of FST288 (*p* < 0.001) (Figure 5). The numbers indicated that the majority of FST315 was released from the hydrogel formulation, contrary to FST288, which was mostly retained in it.

### FST315 and FST288 Do Not Improve Bone Healing in Calvarial Defects, but Show a More Homogeneous Bone Formation Than Controls

**Figure 6:**
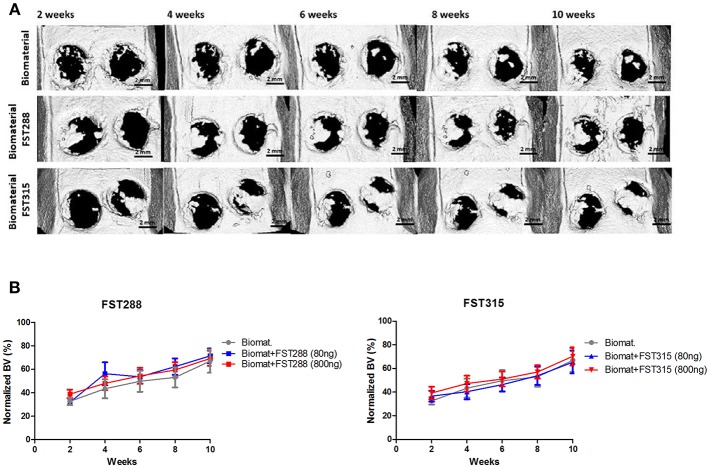
*In vivo* μCT analysis of rat skulls implanted with FST288 and FST315 over time. **(A)** Representative *in vivo* μCT images of the skulls at 2, 4, 6, 8, and 10 weeks after implantation (scale bar: 2 mm) of either biomaterial alone or loaded with FST288 or FST315. In the representative μCT images of both FST variants the right defect was loaded with 800 ng of FST and the left defect was loaded with 80 ng of FST. **(B)** Graphical representations of *in vivo* μCT analysis. Bone volume was normalized to animals without surgical intervention. The effect of the formulation loaded with FST288 (left graph) and FST315 (right graph) was compared to the effect of the use of the biomaterial alone as control group.

Given the promising effects of FST on migration, osteogenesis and vascularization *in vitro*, we decided to investigate whether FST is able to promote bone repair and if there are differences between FST315 and FST288 to induce bone formation. To do so, both FST variants loaded in alginate-MS hydrogel were injected into 5 mm calvarial defects. *In vivo* longitudinal microCT-scans were performed biweekly to monitor the mineralized bone volume (BV) within the defects, and normalized to the BV observed in healthy SD male rats of the same age. At week 2, the positive control with BMP-2 loaded biomaterial exhibited the same amount of bone volume as the healthy animals, which translated into full defect healing at the end of the experiment (Supplementary Figure 2). Implantation of the biomaterial alone led to 66% bone volume compared to healthy animals at the end of the experiment (Figures 6A,B).

No major differences between FST-treated animals and the ones treated with only the biomaterial were observed during 10-weeks follow up. At week 2, 30% of the bone volume observed in healthy animals was already achieved in all the tested conditions. During the time course of the experiment, mild differences in terms of BV were observed between the FST variants. However, at week 10 the overall BV observed was similar in all conditions, varying between 65 and 71% of the defect area (Figures 6A,B).

**Figure 7:**
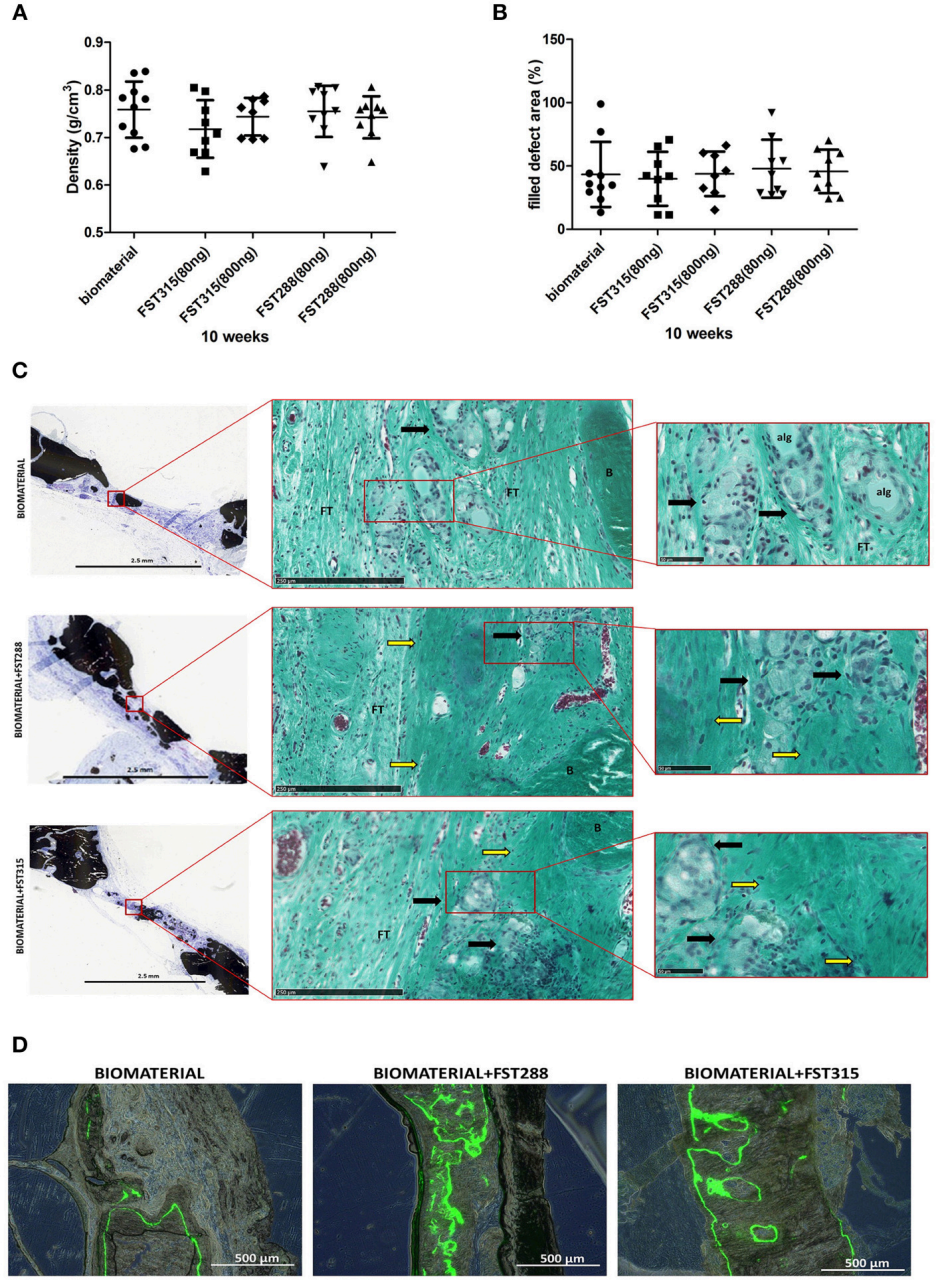
Newly formed bone tissue at 10 weeks of healing. **(A)** Bone density observed by *ex vivo* μCT analysis in the different conditions after implant harvesting. **(B)** Percentage of defect filling by newly formed mineralized tissue analyzed by *ex vivo* μCT. Mean is indicated as the line plotted in the middle of the graphs ± SD. **(C)** Representative pictures of rat skulls implanted with the biomaterial w/o the addition of the FST variants at 10 weeks. Histological analysis includes von Kossa and Goldner's trichrome staining. Von Kossa staining was used to distinguish mineralized tissue (black) (scale bar: 2.5 mm), while Goldner's trichrome staining was used to determine bone histomorphometry. The square grid delimitates the selected magnified area for each image that is shown with Goldner's trichrome staining (scale bars are 250 μm and 50 μm, respectively), showing erythrocytes (red/purple), nuclei (blue/ gray), alginate remains (alg), formed bone **(B)**, and fibrous tissue (FT). Immature ECM is indicated by yellow arrows and regions where the microspheres have been likely degraded are indicated by black arrows. **(D)** Representative fluorescence images of the central region of the explants showing calcein fluorochrome incorporation in the newly formed bone tissue at 10 weeks post-implantation (scale bar: 500 μm) with or without the addition of the FST variants. Fluorescence images are combined with bright-field images of the same area.

The retrieved implants were also scanned *ex vivo*, which allows longer scan times and finer resolution. Bone mineral density of the formed bone did not differ significantly between conditions (Figure 7A) and was comparable to the density found in the calvaria of healthy animals (0.77± 0.03 g/cm^3^, mean ± SD, *n* = 4 animals). The bone coverage of the defect oscillated between 40 and 47% of the total defect area (Figure 7B), which is slightly lower than the coverage found in the *in vivo* longitudinal microCT-scans at 10 weeks. This difference is due to motion artifacts and the settings used in longitudinal microCT-scans on live animals. Either way, microCT-scans showed that bone formation not only occurred around the edge of the defect, but also in the central area. Interestingly, more areas with mineralization were observed in the FST conditions, but overall mineral density did not differ from the controls (Figure 7C). On histology, circular regions were observed in all the conditions. These regions have a similar size to the microspheres used (~50 μm) and in the FST conditions were surrounded by immature ECM, while in the controls, were mostly surrounded by fibrous tissue (Figure 7C). This is more evident when analyzing the mineralization pattern by measuring calcein fluorochrome incorporation. In the controls, the label was mostly present on the outer periphery of the formed bone and hardly any signal was found in the inner side at 4 weeks. In the FST-treated samples the label was found in both periphery and inward area, indicating a broader and more homogeneous mineralization progression (Figure 7D). Besides, in the control samples alginate was still visible, whereas in the FST-treated samples this was undetectable (Figure 7C and Supplementary Figure 3). Histological analysis also showed blood vessels in growth in all the conditions tested (Figure 7C and Supplementary Figure 3).

## Discussion

This study investigated the use of FST for bone repair. We have found that FST was able to recruit and differentiate endothelial cells (ECs), promote cell sprouting and the formation of tube-like structures *in vitro*. Furthermore, FST was also able to recruit osteoprogenitor cells and to enhance committed osteoblast differentiation and mineralization *in vitro*. However, when FST was loaded in our previously developed slow-release formulation (Mumcuoglu et al., 2018) and implanted in a calvarial defect model, bone repair was not improved in 10 weeks' time.

FST is known to be upregulated by migrating ECs (Kozian et al., 1995); however, the chemokinetic effect of follistatin upon ECs was not studied yet. Our results have shown that FST is able to stimulate HUVEC migration when exogenously added to the culture. Furthermore, to assess its effect upon ECs differentiation, we have used two different *in vitro* models-−3D spheroid-sprouting assays and tube-formation assays—to investigate both angiogenesis and vasculogenesis as two of the pivotal processes of the vasculature formation. Interestingly, the formation of tube-like structures seemed to be inversely dose-dependent and FST was able to significantly promote both processes when 28 ng/ml (0.8 nM) was added to the culture in the absence of VEGF or any other co-stimulatory factor. *In vitro* studies have shown that both follistatin and activin are distinctly expressed during the different phases of angiogenesis by bovine aortic ECs (BAECs) and MVECs (Kozian et al., 1995; Glienke et al., 2000). A few studies investigated the effect of activin (25–50 ng/ml) inducing vasculogenesis upon BAECs and sinusoidal ECs (SECs), showing a positive effect when combined with VEGF but contradictory results when used alone to enhance tubulogenesis (Endo et al., 2004; Maeshima et al., 2004). Krneta et al explored the effect of FST and activin in a sprouting angiogenesis assay, showing that FST at 120 ng/ml was able to promote EC sprouting almost at the same level as FGF, while activin addition at 50 ng/ml did not enhance sprouting more than basal control (Krneta et al., 2006). The combination of FGF and activin significantly decreased sprouting (Krneta et al., 2006). *In vivo*, a few papers have shown that FST improved a neovascularization when used on skeletal muscle injury in mice (Zhu et al., 2011) and promoted angiogenesis in the rabbit cornea, especially when combined with FGF (Kozian et al., 1997). It would be interesting to further study this synergistic effect on bone repair. Although we did not specifically study the vascularization processes in our *in vivo* bone defect model, we have found blood vessels in growth in all the treated samples, suggesting that follistatin does not interfere with the natural cascade of events needed for the formation of the vasculature and consequently, it does not have an anti-vasculogenic effect.

Whether FST could attract MSCs and induce bone formation was not studied before. In our *in vitro* study we have demonstrated that FST is able to recruit MSCs. Minor differences were observed between doses, meaning that probably a low dose of FST is enough to promote a chemotactic response upon MSCs. We also investigated the effect of FST upon MSCs and osteoblasts under osteogenic conditions. It is known that FST is highly expressed in developing bone tissues, mainly in osteoblasts (Inoue et al., 1994); however, the effect of FST on osteoblast mineralization was unclear due to conflicting results observed when supplied to mouse and human osteoblast cultures (Abe et al., 2004a; Eijken et al., 2007). In our *in vitro* study, FST further enhanced osteogenic differentiation in committed osteoblasts and not in MSCs.

The stimuli involved in MSC's differentiation to osteoblastic cells may differ from those needed to convert pre-osteoblasts into mature osteoblasts. For example, it is well-known that TGF-β-Smad signaling is crucial in the early phases of osteogenic differentiation, however, it inhibits osteoblast maturation and mineralization (Wu et al., 2016). Consequently, different signal inputs are needed at different stages of the osteoblast differentiation pathway. Our findings suggest that FST's osteogenic effect might be sensitive to which differentiation stage the osteoprogenitor cells are. Previous studies have shown that once FST is synthesized, it remains in the extracellular matrix, exerting an antifibrotic-effect (Nakamura et al., 1991; Maeshima et al., 2014). Indeed, based on previous studies in which have been shown that activin A suppresses osteoblast mineralization by changing the ECM composition and maturity (Eijken et al., 2007; Alves et al., 2013), and in our results, we might conclude that FST is not an osteogenic factor *per se*, but a factor that enhances the mineralization process through its involvement changing the ECM composition while it remains in it. We must remember that FST can only exert its function indirectly, by binding to other molecules and neutralizing their function. Its main antagonist is activin, but FST can also bind to BMPs, with much lower affinity (Glister et al., 2004). Abe et al demonstrated in their study that administration of FST to rat mandibular osteoblasts did not cause significant changes in bone nodule number. BMP-2 facilitated the secretion of FST and this increase in FST interfered with BMP-2 action decreasing bone nodule formation (Abe et al., 2004a). Eijken et al showed in their study that, while FST prevented activin from binding to its receptor, it had no effect on basal or BMP2-induced signaling in human osteoblasts (SV-HFO) (Eijken et al., 2007). Besides, there are several studies that investigated the effect of the addition of activin to osteogenic differentiation of human NHOst cells, showing that its addition strongly inhibited the mineralization process (Eijken et al., 2007; Pearsall et al., 2008). As no receptor has been found for FST, it is widely assumed that FST exerts its regulatory function via antagonizing other proteins with a pivotal role in bone physiology. FST is able to bind almost irreversibly to activin, and with lower affinity to other members of the TGFβ family such as bone morphogenetic proteins. In our *in vitro* experiments, both MSCs and SVHFOs secreted high levels of FST but much lower levels of activin under osteogenic conditions. In fact, the secretion of FST was at least 15-fold higher than the secretion of activin at the same time-points. This amount of FST is much greater than the 2:1 molar ratio needed to neutralize all the activin produced by the cells. The addition of FST to the culture media enhanced mineralization of SVHFOs, but not of MSCs. These findings suggest that FST, even when it is found in a much higher concentration than activin, has a positive effect on osteoblast mineralization but does not affect MSC mineralization. Follistatin can antagonize BMP functions as well as those of the activins, and in view of the sharing of type II receptors between activins and bone morphogenetic proteins (BMPs), further studies should investigate the secretion of these proteins at the different phases of MSC's osteogenic differentiation and the interplay between them.

To investigate the ability of FST to promote bone repair in an orthotopic model, FST was loaded within an *in situ* gelling alginate-based delivery system and implanted in a rat calvarial defect model. We hypothesized that FST, based on previous *in vivo* studies and on our *in vitro* results, may have a positive effect on bone formation. We have used two different FST variants (FST288 and FST315) to assess whether their structural variations -which modulate their properties such as the ability to bind to the cell surface-, could lead to differences between variants in terms of bone repair. In fact, *in vitro* release from the biomaterial showed that during 4 weeks, only 25% of FST288 was released to the medium compared to 80% of FST315.

Two different doses of FST288 and FST315 were loaded within the biomaterial to elucidate if there is a limiting concentration of FST that leads to bone repair enhancement and whether FST excess prevents bone repair. FST doses used (800 ng and 80 ng per implant) were based on both our *in vitro* studies and the literature. Serum levels of FST are found in the ng range in mice (Barakat et al., 2008), and our results show that the lowest dose of FST used in the *in vitro* experiments (28 ng/ml) improved crucial processes involved in bone formation such as cell migration, osteogenesis, and neovascularization. However, none of the variants improved bone formation compared to the biomaterial in 10 weeks' time. Besides, during the degradation of the biomaterial the protein is released and the differences in FST concentrations of the separate defects may become minor.

Timing of secretion of FST during bone repair seems crucial. When a demineralized matrix was subcutaneously implanted in rats, FST was highly expressed during the initial stages of osteogenesis but decreased along with differentiation (Funaba et al., 1996). Injections of FST in the implants 10 days after implantation resulted in lower calcium content in the implants suggesting that the endochondral ossification process was retarded or inhibited (Funaba et al., 1996). Nagamine et al showed that neither FST nor activin were expressed in osteogenic cells at the periosteum or at the cortical bone in the intact femurs of the rat (Nagamine et al., 1998). Interestingly, in a fractured-femur FST was highly expressed during the first stages of bone healing in osteogenic cells, as well as in the ECM of the periosteum and proliferating chondrocytes, while activin A was almost undetectable in those regions. Furthermore, Activin A was detected especially around osteoclast-like cells on the surface of the newly-formed trabecular bone (Nagamine et al., 1998), which is in line with previous publications (Inoue et al., 1994). Altogether indicates that the expression of FST during different phases of both intramembranous and endochondral ossification is important. However, the role of FST in bone repair has been investigated either using ectopic models or long bone fracture models (Funaba et al., 1996; Nagamine et al., 1998) in which the mechanisms of bone formation differ considerably from the events taking place in the development of the flat bones of the skull. It is difficult to compare *in vitro* and *in vivo* studies, but our *in vitro* experiments have shown that FST effects do not respond to a classical dose-response curve; MSC migration and mineralization may need different FST doses that may not fit with FST release timing and dosage from the biomaterial chosen in this study. In fact, when the alginate formulation was implanted alone, residual alginate was still visible 10 weeks post implantation, whereas in the FST-treated samples this phenomenon was not detected. In FST-treated samples mineralization occurred both on the outer and inner area of the defect, contrary to what was observed in the controls, where calcein was mostly incorporated on the outer periphery of the defect. An alginate-based formulation was used as FST carrier to achieve FST's slow release, but FST appears to be an early player in bone formation. As we have previously mentioned, our data and the literature suggest that FST exerts its function through the ECM, enhancing its mineralization. The slow release alginate-based system may not be optimal for bone repair when loaded with FST and might influence the FST effect upon ECM mineralization due to a suboptimal FST release timing. Alginate has been widely used for bone tissue engineering due to its biocompatibility, easy handling and degradation properties (Venkatesan et al., 2015). We have previously used RCP encapsulated in an injectable alginate hydrogel ectopically, and the formulation releasing BMP-2 effectively promoted bone formation. However, when used in bone defect repair, alginate seemed to not only delay the protein release, but also cell and cytokine infiltration within the defects (Mumcuoglu et al., 2018). Activin A has been identified as a pivotal molecule during the initial inflammatory response (Hedger et al., 2011), which can be also provoked by surgical stress. Interestingly, FST increased in the circulation several hours after activin A and it is believed that the increase observed in FST levels was partly responsible for the clearance of activin A from the bloodstream (Hedger et al., 2011). In our study, alginate, due to its chemostatic effect, could have prevented or delayed the cascade of events needed for bone repair such as the influx of inflammatory cells and growth factors, or the clearance of activin A from the injury site. Bleeding could also have affected the biomaterial physical-chemical properties. Certainly, it would be interesting to assess whether FST addition shows a positive effect in a bone defect when used in a different type of delivery system with a faster release. The affinity to bind cell surface receptors dictates the main biological action of FST. FST315 is considered to act more in an endocrine fashion whereas FST288 does it in an autocrine manner. In fact, FST315 has been used systemically as a therapeutic agent to treat skeletal muscle diseases: however, it exhibits rapid clearance kinetics. Consequently, newly engineered FST315 variants with improved pharmacokinetic properties have been developed, showing promising results in the treatment of several musculoskeletal injury models (Datta-Mannan et al., 2013; Yaden et al., 2014). In our study we aimed to enhance *in situ* bone formation and in the view of our release studies and the greater affinity for cell surface proteoglycans of FST288, the use of this FST variant in a rapid release system is intriguing, and could prompt the assessment of new strategies in bone defect repair.

To our knowledge, this is the first study that instead of focusing in osteogenic differentiation to investigate FST possible role in bone formation, assesses the effect of FST upon chemotaxis and vasculogenesis as well due to their essential role in bone formation, and directly evaluates its possible influence in an orthotopic bone defect such as calvarial-defect. FST plays an important role in bone metabolism, mostly acting as activin controller but also regulating the function of other members of the TGFβ family (Funaba et al., 1996; Gajos-Michniewicz et al., 2010). However, FST action spans so many different processes that its effect in particular cellular events involved in bone formation was unclear. In summary, this study has shown that FST is able to stimulate cell recruitment, vasculogenesis, and osteogenesis; vital processes for a successful bone regeneration. Using the calvarial defect model we could not show a clear improvement in bone repair with FST -which can be due to several causes such as poor bone-forming capacity, underdosage and suboptimal release kinetics among others-, though we observed a more homogeneous mineralization. There is still a lack of knowledge about the role of FST in the acute phase reaction and the effect of its administration in the early phases of bone repair. Therefore, to move on further using growth factor-based therapies, mechanistic approaches should be taken in consideration to investigate how and in which extent FST, as well as its interaction with other proteins, such as activins and BMPs, regulates key processes in bone metabolism and repair. Besides, the optimal release kinetics of FST must be investigated *in vivo* to successfully translate its use into bone tissue engineering based therapies.

## Data Availability

The datasets generated for this study are available on request to the corresponding author.

## Author Contributions

SF-G, EF, GvO, and MvD: Conceptualization and Investigation; SF-G, JW-B, and IR: Formal analysis; GvO, SK, and JvL: Funding acquisition; SF-G, EF, MvD, JW-B, IR, MS, DM, BvdE, GvO, and JvL: Methodology; SF-G, EF, and MvD: Project administration; GvO, HW, SK, and BvdE: Resources; EF, HW, GvO, JvL, and MvD: Supervision; JW-B and IR: Validation; SF: Writing—original draft; EF, BvdE, GvO, JvL, and MvD: Writing – review & editing.

### Conflict of Interest Statement

SK is employed by Fujifilm. The remaining authors declare that the research was conducted in the absence of any commercial or financial relationships that could be construed as a potential conflict of interest.
