# Supplementary material for: Follistatin Effects in Migration, Vascularization, and Osteogenesis in vitro and Bone Repair in vivo
Source: Front Bioeng Biotechnol. 2019 Mar 1;7:38. doi: 10.3389/fbioe.2019.00038 (PMC6405513; doi:10.3389/fbioe.2019.00038)
Supplement: Supplementary file 1 [file Data_Sheet_1.docx]

Supplementary Material

Follistatin effects in migration, vascularization and osteogenesis *in vitro* and bone repair *in vivo*

Shorouk Fahmy-Garcia ^1,3^, Eric Farrell ^2^*, Janneke Witte-Bouma ^2^, Iris Robbesom-van den Berge ^3^, Melva Suarez ^4^, Didem Mumcuoglu ^1,6^, Heike Walles ^4^, Sebastiaan G.J.M. Kluijtmans ^6^, Bram C.J. van der Eerden ^3^, Gerjo J.V.M. van Osch ^1,5^, Johannes P.T.M. van Leeuwen ^3^, Marjolein van Driel ^3^

*** Correspondence:** Eric Farrell. e.farrell@erasmusmc.nl

## Supplementary Figures


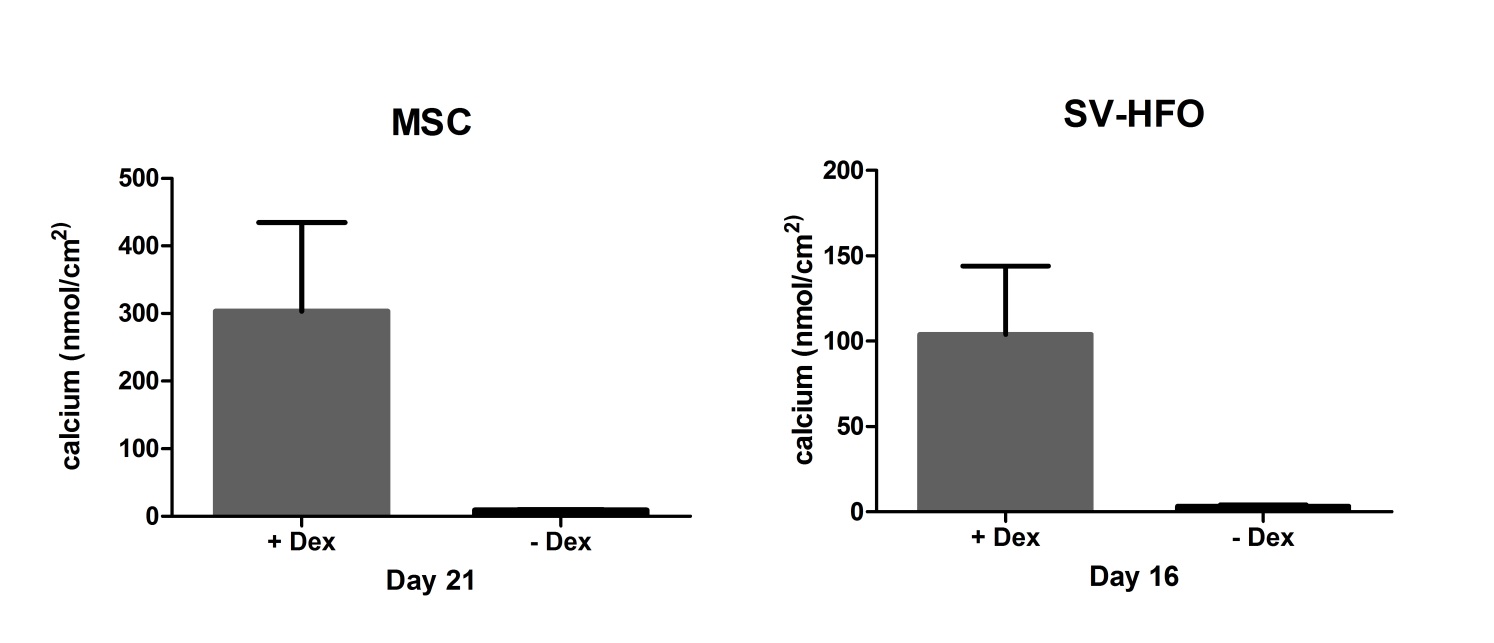


**Supplemental Figure 1. Calcium deposition in MSC and SV-HFO culture in the presence and absence of dexamethasone.** Human MSCs and osteoblasts were induced to mineralize using medium consisting in DMEM High Glucose with 10% FCS, 1.5 µg/mL fungizone, 50 µg/mL gentamicin, 25 µg/mL ascorbic acid-2-phosphate, and 10 mM β-glycerophosphate in the absence or continuous presence of dexamethasone. Quantification of calcium deposition (nmol/ cm^2^) in the MSC and SV-HFO extracellular matrix at day 21 and day 16 showed that calcium deposits were only detected in the presence of dexamethasone (n=1 donor performed in triplicate). The bars show the mean ± SD.

**
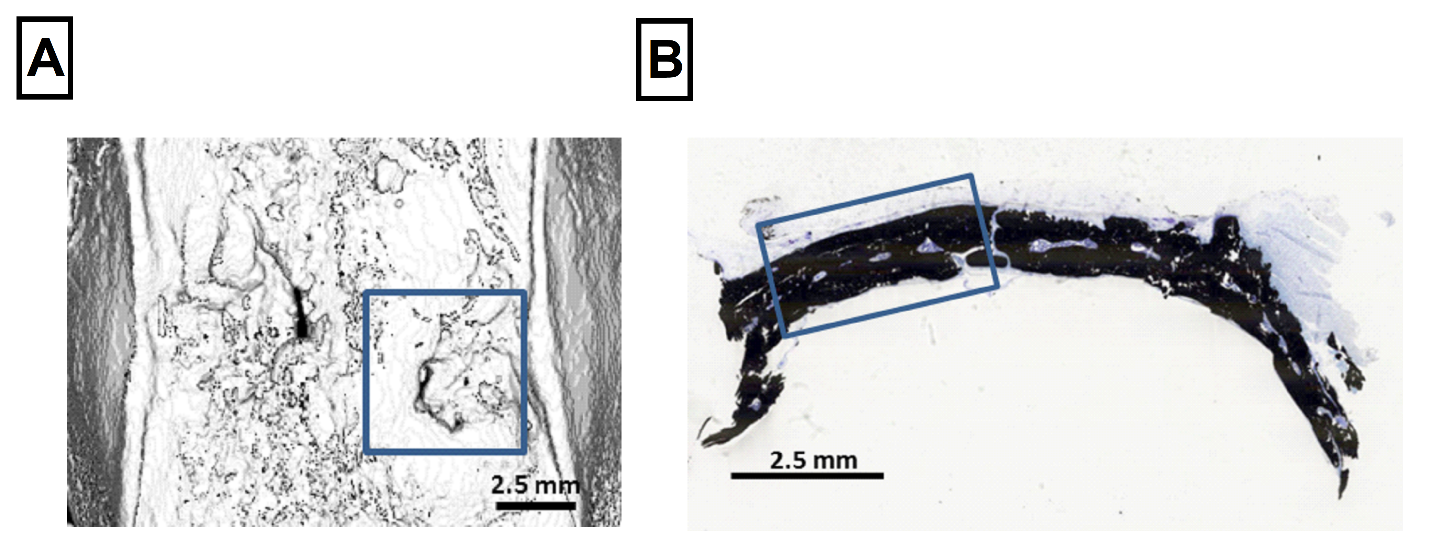
Supplemental Figure 2. Bone regeneration in calvarial defects with 2 µg of BMP-2 loaded in the formulation.** A. μCT analysis indicated healed defects, bridged by mineralized bone tissue. B. Representative histological section stained with von Kossa. The square indicates the approximate area of the defect. Scale bars: 2.5 mm.

**
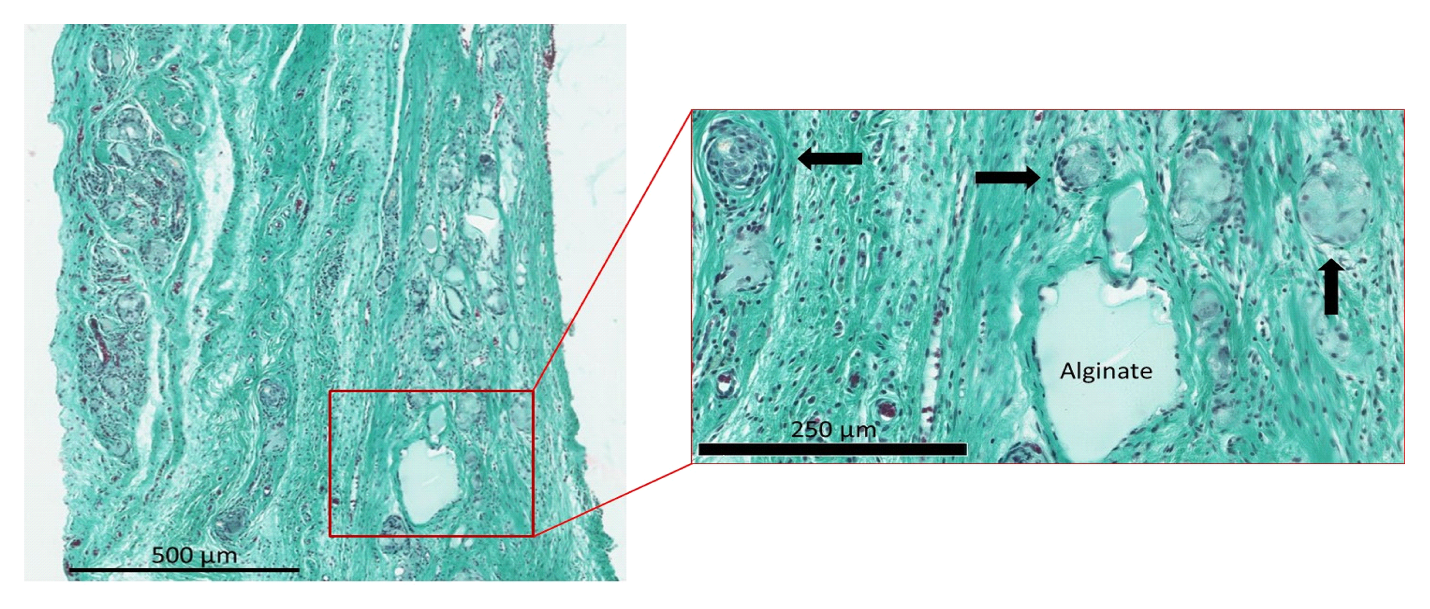
**

**Supplemental Figure 3. Residual alginate was found in the bone defects 10 weeks post-implantation in the control-treated samples.** Samples were stained with Goldner´s trichrome (scale bars: 500 μm). The square grid delimitates a magnified area (scale bar: 250 μm) in which areas with alginate are found. The black arrows indicate regions where the microspheres might have been degraded and cell-infiltration has occurred.
